# Successful Management of a Large Orbital Hemangioma in an Infant: A Case Report

**DOI:** 10.7759/cureus.58062

**Published:** 2024-04-11

**Authors:** Eman Abdulla, Zainab Abdulla, Warda Alalawi, Shaikha Fathalla, Farah Asad

**Affiliations:** 1 General Practice, Mansoura University, Mansoura, EGY; 2 General Practice, First Moscow State Medical University, Moscow, RUS; 3 General Practice, Almoosa Specialist Hospital, Al Khobar, SAU

**Keywords:** propranolol therapy, infant, magnetic resonance imaging, orbital hemangioma, eyelid swelling

## Abstract

Orbital hemangiomas are benign vascular tumors commonly affecting infants and young children, often manifesting with proptosis, eyelid swelling, or discoloration. Propranolol has emerged as the primary therapy due to its efficacy in promoting regression and minimizing complications. Here, we present a case of a previously healthy six-month-old male infant with progressive right eyelid swelling and discoloration. Magnetic resonance imaging confirmed a large orbital hemangioma. The patient was referred to an ophthalmology center where treatment with propranolol resulted in substantial improvement. Early recognition and initiation of propranolol therapy are crucial in managing orbital hemangiomas in pediatric patients. This case underscores the successful outcome achievable with pharmacologic intervention and emphasizes the importance of long-term follow-up for monitoring and optimizing patient outcomes.

## Introduction

Orbital hemangiomas are benign vascular tumors that commonly affect infants and young children [1]. They typically present with a gradual onset of proptosis, eyelid swelling, or discoloration, which can lead to significant functional and cosmetic concerns [2]. While most orbital hemangiomas are self-limiting and undergo spontaneous regression, large or symptomatic lesions may require intervention to prevent visual impairment or disfigurement [2,3]. Propranolol, a non-selective beta-blocker, has emerged as a first-line treatment for infantile hemangiomas due to its efficacy in promoting regression and minimizing potential complications [2]. Despite its widespread use, the precise mechanisms underlying the therapeutic effects of propranolol on orbital hemangiomas remain incompletely understood [2,4]. Furthermore, optimal dosing regimens, duration of treatment, and long-term outcomes of propranolol therapy in this population are areas of ongoing research and debate [1-5]. In this context, we present a case of a six-month-old infant with a large orbital hemangioma treated with propranolol, highlighting the clinical presentation, diagnostic work-up, management, and long-term follow-up of this rare but clinically significant condition.

## Case presentation

A previously healthy six-month-old male infant presented to the pediatric clinic with a chief complaint of right eyelid swelling and discoloration. According to the caregiver, the swelling had been progressively increasing over the past few weeks, accompanied by mild discomfort on palpation. There was no history of trauma, fever, or other systemic symptoms noted by the caregiver. The infant's medical history was unremarkable, with no significant past illnesses or surgeries reported. Family history was non-contributory for any ocular or systemic disorders. The infant was born at full term via uncomplicated vaginal delivery and had achieved appropriate developmental milestones.

**Figure 1:**
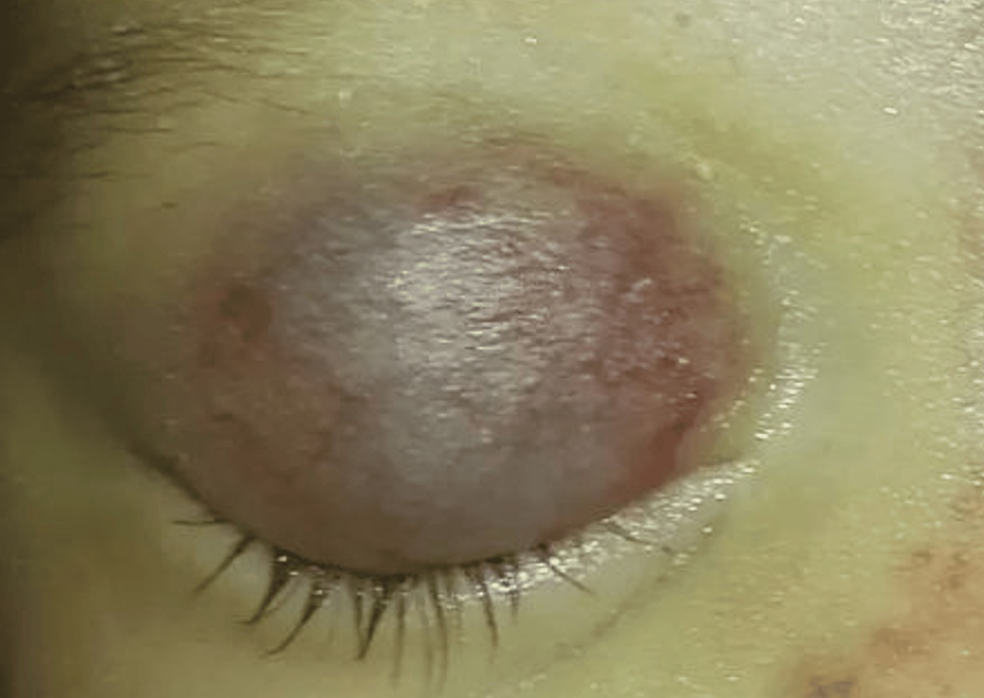
Clinical photograph of the right eye on presentation. This photograph depicts the right eye at presentation, demonstrating pronounced swelling and discoloration of the eyelid, indicative of orbital hemangioma. Note: Only the right eye is shown in the image to maintain confidentiality, as per the parent's request.

Upon physical examination, the infant was alert, responsive, and in no acute distress. Vital signs were as follows: heart rate of 130 beats per minute, respiratory rate of 30 breaths per minute, blood pressure of 80/50 mmHg, and temperature of 37.0°C. Inspection of the right eye revealed marked swelling and erythema involving the upper eyelid, extending to the lateral canthus (Figure 1). The affected area appeared warm to touch, with no evidence of fluctuance or discharge. Visual acuity in both eyes was intact, and extraocular movements were preserved. The remainder of the ocular examination was unremarkable, with no evidence of proptosis, globe displacement, or abnormal pupil reactions.

**Figure 2:**
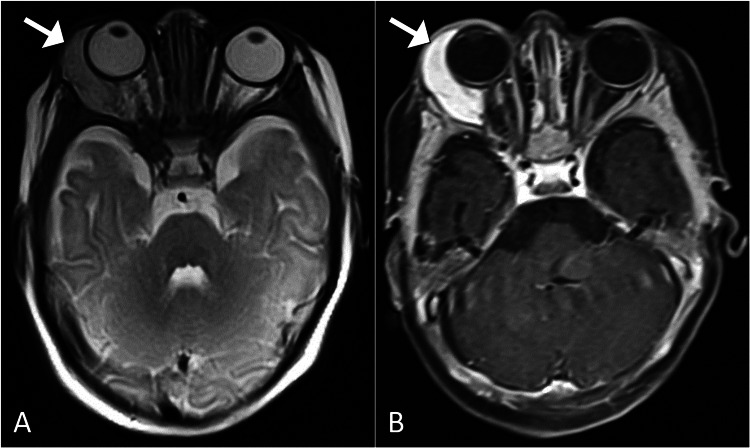
Magnetic resonance imaging of the brain and orbits T2-weighted (A) and T1-weighted post-contrast (B) magnetic resonance images of the orbits, illustrating a distinct, homogenously enhancing right orbital lesion (arrow) consistent with an orbital hemangioma.

Given the concerning presentation, further work-up was pursued to elucidate the underlying etiology of the eyelid swelling. Initial laboratory investigations were within normal limits, including complete blood count, comprehensive metabolic panel, and coagulation studies (Table 1). Imaging studies were subsequently performed to assess the extent and nature of the lesion. Magnetic resonance imaging of the orbit revealed a well-defined, homogenously enhancing mass located within the right orbital space, consistent with the diagnosis of orbital hemangioma. The lesion was noted to be large, exerting a mass effect on surrounding structures without evidence of invasion into adjacent tissues (Figure 2).

**Table 1:** Laboratory investigations and results. This table provides a comprehensive overview of the patient's laboratory investigations, demonstrating that all parameters fell within normal ranges.

Laboratory test	Value	Reference range
Hemoglobin	12.5 g/dL	11.0 - 14.0 g/dL
White blood cell count	8.2 x 10^3^/μL	5.0 - 17.0 x 10^3^/μL
Platelet count	250 x 10^3^/μL	150 - 450 x 10^3^/μL
Sodium	138 mmol/L	135 - 145 mmol/L
Potassium	4.2 mmol/L	3.5 - 5.0 mmol/L
Chloride	102 mmol/L	95 - 105 mmol/L
Bicarbonate	24 mmol/L	22 - 30 mmol/L
Blood urea nitrogen	12 mg/dL	5 - 20 mg/dL
Creatinine	0.6 mg/dL	0.4 - 1.2 mg/dL
Glucose	95 mg/dL	70 - 100 mg/dL
Prothrombin time	12 seconds	11.0 - 13.0 seconds
International normalized ratio	1.0	0.8 - 1.2
Activated partial thromboplastin time	30 seconds	25 - 35 seconds

Upon confirmation of the diagnosis, the infant was promptly referred to the ophthalmology center for further evaluation and management. After a thorough discussion with the caregivers regarding treatment options and potential risks, a decision was made to initiate pharmacologic therapy with propranolol. The infant was closely monitored for hemodynamic changes and adverse effects during titration. Over the subsequent weeks, there was a noticeable improvement in the size and color of the eyelid swelling, accompanied by the resolution of associated symptoms.

**Figure 3:**
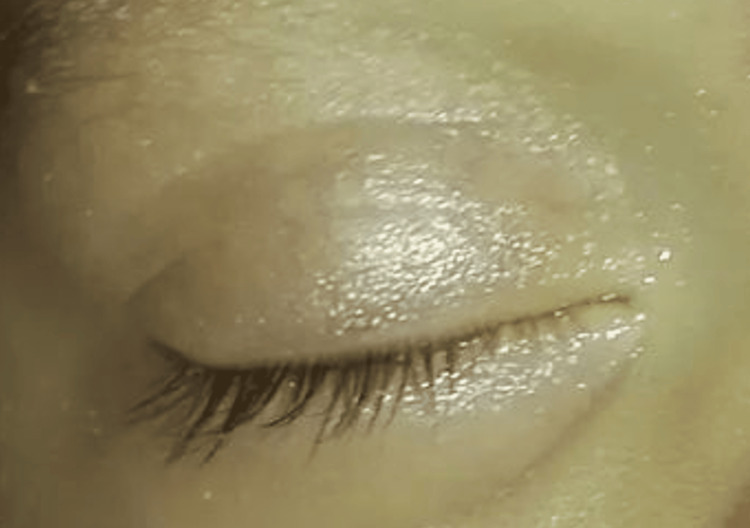
Clinical photograph of the right eye post treatment. This photograph showcases the right eye post treatment, revealing complete resolution of the previously observed swelling and discoloration following successful propranolol therapy. Note: Only the right eye is shown in the image to maintain confidentiality, as per the parent's request.

At the three-month follow-up appointment, the infant demonstrated complete resolution of the eyelid swelling and discoloration, with no residual symptoms or signs of orbital hemangioma recurrence (Figure 3). Regular follow-up appointments were scheduled to monitor long-term outcomes and ensure appropriate management of the orbital hemangioma.

## Discussion

The management of orbital hemangiomas in pediatric patients poses a clinical challenge due to the potential for visual impairment and disfigurement. Our case underscores the importance of early recognition, prompt referral to specialized centers, and initiation of appropriate therapy to optimize outcomes in affected infants. The diagnosis of orbital hemangiomas relies primarily on clinical presentation and imaging studies [2,4]. Classic features include progressive eyelid swelling, proptosis, and palpable mass, as demonstrated in our case. Imaging modalities such as orbital ultrasound and magnetic resonance imaging play a crucial role in confirming the diagnosis, delineating the extent of the lesion, and assessing its relationship to surrounding structures [3-6].

Propranolol has emerged as a cornerstone of pharmacologic therapy for infantile hemangiomas, including those affecting the orbit. Its mechanism of action in hemangioma regression is multifaceted, involving vasoconstriction, inhibition of angiogenesis, and induction of apoptosis in proliferating endothelial cells [3-6]. The favorable safety profile and high efficacy of propranolol have led to its widespread adoption as first-line therapy, as evidenced by the significant improvement observed in our case following initiation of treatment.

Optimal dosing regimens and duration of propranolol therapy in the management of orbital hemangiomas remain areas of active investigation [2,5]. While the standard protocol involves oral administration of propranolol at a dose of 2-3 mg/kg/day divided into two or three doses, individualized approaches may be necessary based on the patient's age, weight, comorbidities, and treatment response [1,6]. Close monitoring for adverse effects, particularly hypoglycemia, bradycardia, and hypotension, is paramount during the titration period and throughout therapy [2,5].

Long-term follow-up is essential to monitor for recurrence, assess cosmetic outcomes, and evaluate potential late effects of propranolol therapy [3]. Although most orbital hemangiomas demonstrate spontaneous regression with or without intervention, vigilance is warranted to detect any residual or recurrent disease early [3,5]. Our case highlights the importance of regular clinical evaluations and imaging studies to ensure sustained resolution and optimal visual outcomes in affected infants.

## Conclusions

In conclusion, our case underscores the efficacy of propranolol as a primary therapeutic modality in the management of large orbital hemangiomas in pediatric patients. Early recognition, prompt referral to specialized centers, and initiation of appropriate therapy are crucial in optimizing outcomes and minimizing potential complications associated with this condition. The favorable safety profile and high efficacy of propranolol make it an attractive option for achieving regression of orbital hemangiomas while preserving visual function and cosmetic appearance. Long-term follow-up is essential to monitor for recurrence, assess treatment response, and ensure sustained resolution of the lesion. Continued research efforts are warranted to further elucidate the mechanisms of action of propranolol, refine treatment protocols, and optimize long-term outcomes in this challenging patient population.
